# Immunohistochemical expression of NRF2 is correlated with the magnitude of inflammation and fibrosis in chronic liver disease

**DOI:** 10.1002/cam4.6538

**Published:** 2023-09-21

**Authors:** Keii To, Kosuke Okada, Takahisa Watahiki, Hideo Suzuki, Kiichiro Tsuchiya, Katsutoshi Tokushige, Masakazu Yamamoto, Shun‐ichi Ariizumi, Junichi Shoda

**Affiliations:** ^1^ Department of Gastroenterology, Institute of Medicine University of Tsukuba Ibaraki Japan; ^2^ Doctoral Program in Medical Sciences, Graduate School of Comprehensive Human Sciences University of Tsukuba Ibaraki Japan; ^3^ Division of Medical Sciences, Institute of Medicine University of Tsukuba Ibaraki Japan; ^4^ Institute of Gastroenterology and Internal Medicine Tokyo Women's Medical University Tokyo Japan; ^5^ Department of Surgery, Institute of Gastroenterology Tokyo Women's Medical University Tokyo Japan

**Keywords:** chronic liver disease, hepatocellular carcinoma, KEAP1, NASH, NRF2

## Abstract

**Background:**

The nuclear factor E2‐related factor 2–Kelch‐like Ech‐associated protein (NRF2–KEAP1) pathway is a major cellular defense mechanism against oxidative stress. However, the role of NRF2–KEAP1 signaling in the development of chronic liver disease remains unclear.

**Methods:**

Clinical liver specimens from 50 hepatocellular carcinoma (HCC) developed from non‐alcoholic steatohepatitis (NASH), 49 HCCs developed from chronic viral hepatitis C (CHc), and 48 liver metastases of colorectal cancer (CRC) from both tumorous and non‐tumorous areas were collected during hepatic resection surgery. They were evaluated by immunohistochemical analyses of hematoxylin–eosin, Masson's trichrome, NRF2, and KEAP1, and compared with clinicopathological information.

**Results:**

Hepatic inflammation and fibrosis were more severe in the low‐intensity NRF2 group than in the high‐intensity NRF2 group both between CRC and NASH (Low vs. High: inflammation; *p* = 0.003, fibrosis; *p* = 0.014), and between CRC and CHc (Low vs. High: inflammation; *p* = 0.031, fibrosis; *p* = 0.011), which could indicate that NRF2 expression in cytosol of hepatocytes was inversely correlated with liver inflammation and fibrosis in non‐tumorous areas. The dense staining of NRF2 in the nuclei of non‐tumor hepatocytes positively correlated with liver inflammation (CRC and NASH; *R* = 0.451, *p* < 0.001, CRC and CHc; *R* = 0.502, *p* < 0.001) and fibrosis (CRC and NASH; *R* = 0.566, *p* < 0.001, CRC and CHc; *R* = 0.548, *p* < 0.001) in both NASH and CHc, and was inversely correlated with hepatic spare ability features such as platelet count (*R* = −0.253, *p* = 0.002) and prothrombin time (*R* = −0.206, *p* = 0.012). However, KEAP1 expression was not correlated with NRF2 expression levels and nuclear staining intensity.

**Conclusions:**

Nuclear translocation of NRF2 was correlated with the magnitude of liver inflammation and fibrosis in chronic liver disease. These results suggest that NRF2 plays a protective role in the development of chronic liver diseases such as NASH and CHc.

## 1 INTRODUCTION

Hepatocellular carcinoma (HCC) is a common cancer type and one of the leading causes of cancer mortality worldwide.^1^, ^2^ Chronic viral hepatitis C (CHc) plays a major role in HCC^3^ by promoting liver cirrhosis and tumorigenesis. In addition, evidence has accumulated showing that obesity and diabetes increase the risk of HCC both worldwide^4^ and in Japan.^5^, ^6^ Obesity and diabetes cause non‐alcoholic fatty liver disease, which can develop into non‐alcoholic steatohepatitis (NASH), a progressive liver disease characterized by steatosis, inflammation, and fibrosis that leads to liver cirrhosis and HCC.^7^, ^8^ The development of NASH and HCC is associated with multiple parallel factors, in a theory called “Multiple parallel hits theory,”^9^ Insulin resistance,^8^ endoplasmic reticulum stress,^10^ lipopolysaccharide derived from the intestines,^11^ and oxidative stress^12^, ^13^ are associated with hepatocarcinogenesis through the modulation of many cancer driver genes and cancer pathway genes,^14^, ^15^ Nuclear factor E2‐related factor 2 (NRF2), a transcriptional factor, is a master regulator of the cellular adaptive response to oxidative stress.^16^ NRF2 is sequestered in the cytosol by Kelch‐like Ech‐associated protein (KEAP1). Upon oxidative challenge, modification of KEAP1 sulfhydryl groups results in the stabilization and nuclear translocation of NRF2.^17^ In previous studies conducted in *Nrf2*‐knockout mice and *Keap1*‐knockdown mice in which NRF2 was constitutively activated, we reported that NRF2 had protective roles against NASH through the inhibition of oxidative stress and fibrosis in the liver.^18^ Moreover, loss of KEAP1 activity through somatic mutations has been reported in HCC,^14^, ^15^, ^19^ indicating that constitutive activation of NRF2 and aberrant NRF2 activation causes chemotherapeutic resistance in HCC^19^ and other cancers.^20^ However, the behavior and role of NRF2 in chronic liver disease was unclear because of the difficulty in liver sampling, especially non‐tumorous areas including liver cirrhosis.

The aim of the present study was to explore the role of NRF2 in chronic liver diseases including NASH and CHc, as well as in HCC, through immunohistochemical analyses of clinical liver specimens and comparisons with clinicopathological information. We demonstrated that the expression and nuclear translocation of NRF2 in hepatitis and cirrhosis were correlated with liver inflammation and fibrosis. These results suggest that NRF2 might have a protective role against the development of chronic liver disease.

## 2 METHODS

### 2.1 Patients

We conducted a cross‐sectional study using data collected from 147 individuals who underwent liver resection surgery for HCC following NASH (NASH group; *n* = 50), chronic viral hepatitis C (CHc group; *n* = 49), and liver metastasis from colorectal cancer (CRC group; *n* = 48) at Tokyo Women's Medical University Hospital between May 10, 2010 and March 12, 2019. Patients with a tumor size of <5 cm were included in this study. Patients with tumor invasion of portal or hepatic veins, direct invasion of adjacent organs, and/or extrahepatic metastasis were excluded. Patients who received trans‐arterial chemoembolization, radiofrequency ablation, or direct acting antivirals for CHc were not included in this study. It was difficult to follow‐up the prognosis of the patients because of the short period after surgery. Moreover, many patients were referred to other hospitals after their surgery.

### 2.2 Histological analysis

Liver tissue specimens from each patient were fixed in 10% buffered formalin and processed by standard histological techniques. Slides were stained with hematoxylin & eosin (HE) and Masson's trichrome using standard protocols. To evaluate the histopathological severity of chronic liver disease in non‐tumorous areas, we defined non‐tumorous areas of CRC as the control. The steatosis, activity, and fibrosis (SAF) score was assessed for the grade of steatosis (0–3), activity (perilobular inflammation, 0–2; ballooning, 0–2; total, 0–4), and stage of fibrosis (0–4), and was compared in the CRC and NASH groups,^21^ while the New Inuyama classification was assessed for the grade of activity (0–3) and stage of fibrosis (0–4), and compared in the CRC and CHc groups.^22^

**FIGURE 1:**
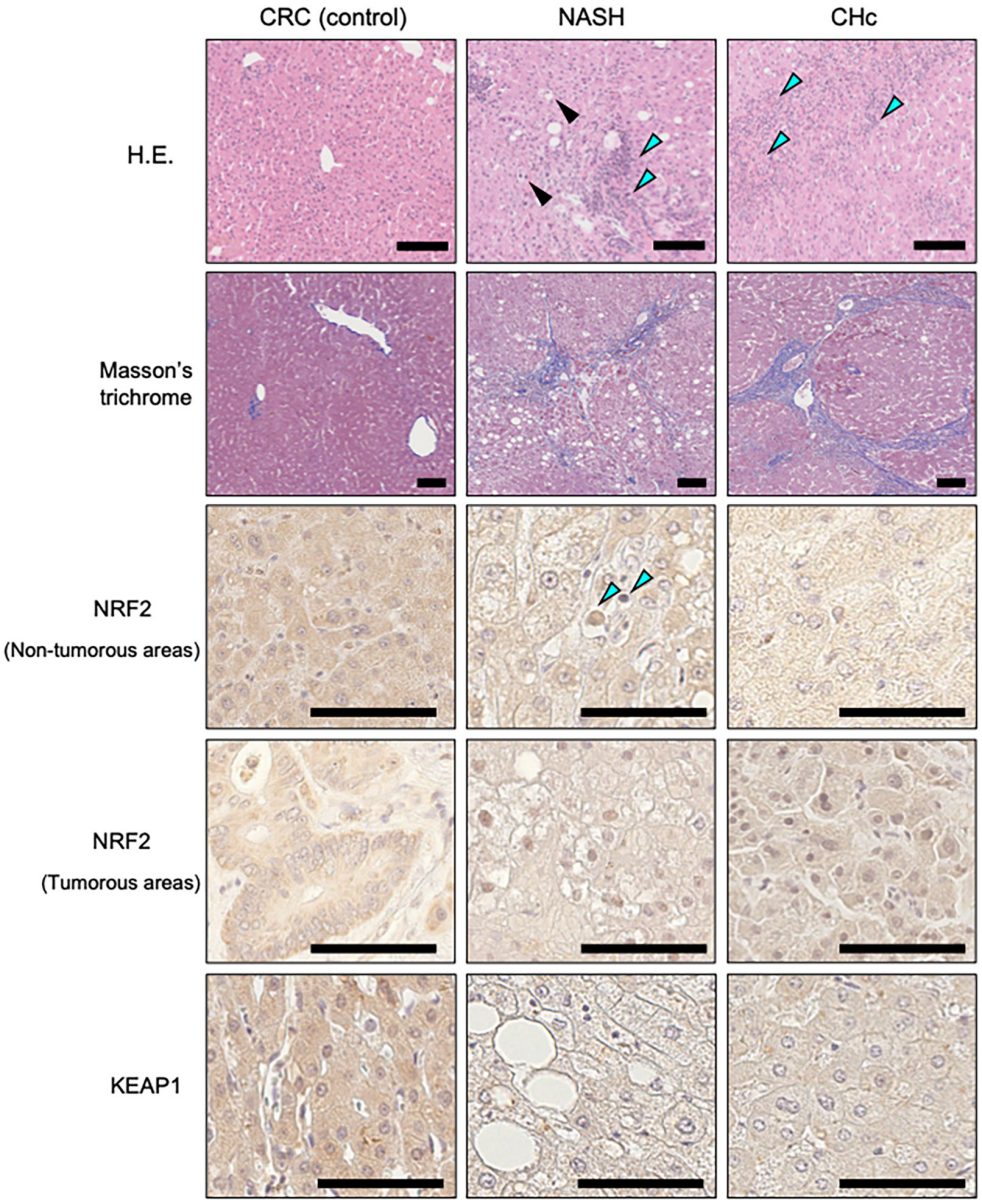
Histopathological staining of non‐tumorous and tumorous areas. HE‐stained sections were analyzed for liver steatosis and inflammation in non‐tumorous areas. Blue arrows indicate inflammatory cell infiltration (perilobular inflammation) and the black arrow indicates a ballooning cell. Masson's trichrome‐stained sections were analyzed for liver fibrosis in non‐tumorous areas. NRF2 was mainly stained and expressed in the cytosol of hepatocytes in both non‐tumorous and tumorous areas. Regarding the intensity of cytosolic NRF2 expression, the patient with CRC was classified as high expression, the patient with NASH was classified as medium, and the patient with CHc was classified as low. Cells in hepatocellular carcinoma exhibited dense nuclear staining of NRF2 in tumorous areas. Regarding the intensity of cytosolic KEAP1 expression, the patient with CRC was classified as high and the patient with NASH/CHc was classified as low. Blue arrows indicate dense nuclear staining of NRF2 in non‐tumorous areas, and many HCC cases had dense immunostaining of NRF2 in the nucleus, indicating NRF2 translocation into the nucleus. All scale bars are 100 μm. CHc, chronic viral hepatitis C; CRC, colorectal cancer; HCC, hepatocellular carcinoma; KEAP1, Kelch‐like Ech‐associated protein; NASH, non‐alcoholic steatohepatitis; NRF2, nuclear factor E2‐related factor 2.

**FIGURE 2:**
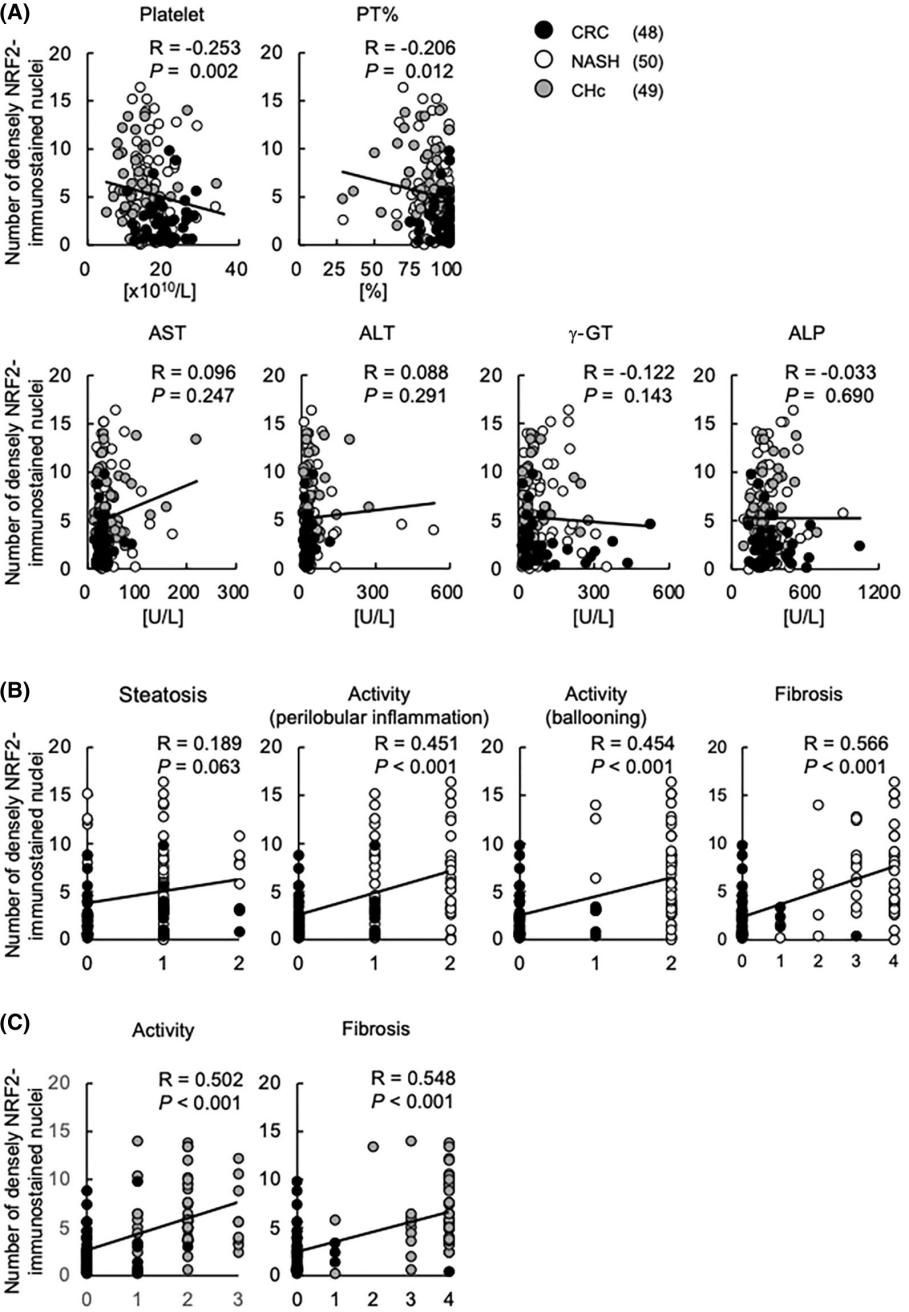
Dense immunostaining of NRF2 in the nuclei of hepatocytes is associated with liver inflammation and fibrosis in non‐tumorous areas. The number of densely NRF2‐immunostained hepatocyte nuclei was analyzed and compared with blood biochemistry (A), histopathology in non‐tumorous areas by SAF score in CRC and NASH (B), and histopathology in non‐tumorous areas by the New Inuyama classification in CRC and CHc (C) using dot plots. CHc, chronic viral hepatitis C; CRC, colorectal cancer; NASH, non‐alcoholic steatohepatitis; NRF2, nuclear factor E2‐related factor 2; SAF, steatosis, activity, and fibrosis.

For experiments on immunohistochemical expression and localization of NRF2, liver tissue sections were immunostained with NRF2 polyclonal antibody (ab31163; Abcam). To evaluate the relationship between NRF2 immunostaining and liver pathophysiology in non‐tumorous areas, the patients were divided into three groups according to NRF2 expression in cytosol of hepatocytes: low, medium, and high (Figure 1; Table 3). Furthermore, they were divided into two groups according to whether their localization was cytosolic only or cytosolic plus nuclear (+ nucleus group; Table 4). Moreover, to clarify the nuclear translocation of NRF2 in the liver pathophysiology of non‐tumorous areas, namely NASH and/or CHc severity, the number of nuclei densely stained for NRF2 was quantified by counting 150 cells in five fields of view at ×40 magnification and compared with clinicopathological information (Figure 2). Furthermore, because HCC exhibited dense nuclear NRF2 immunostaining, the patients were divided into three groups: low (<33%), medium (33%–66%), and high (>66%; Table S1, lower panel).

To examine KEAP1 expression, liver tissue sections were immunostained with KEAP1 polyclonal antibody (10503‐2‐AP; Proteintech Group, Inc). KEAP1 was expressed in the cytosol of hepatocytes in both non‐tumorous and tumorous areas (Figure 1). Moreover, the staining was uniform and the difference was smaller compared with NRF2; hence, the patients were divided into two groups according to the intensity of cytosolic KEAP1 expression: low or high in non‐tumorous areas (Table 5), and low or high in HCC areas (Table S2). In addition, the expression of KEAP1 was compared with the intensity and localization of NRF2 because KEAP1 binds with NRF2 under normal conditions and inhibits NRF2 function (Figure S1). The immunostained slides without primary antibodies were used as negative controls in the immunohistochemical analyses.

Images were observed and made by NDP.view2 virtual slide system (Hamamatsu Photonics K.K). In this study, the same pathologist examined the slides three times in a blinded fashion to account for the potential sources of variation.

### 2.3 Lifestyle‐related disease definitions and clinical laboratory measurements

Hypertension (HT), diabetes mellitus (DM) type 2, and dyslipidemia (DL) were identified through medical history examination and/or intervention.

Biochemical analyses of clinical and laboratory measurements were performed by the Japan Society of Clinical Chemistry transferable method, the enzymatic method, and the direct method at Tokyo Women's Medical University Hospital as a preoperative assessment. The fibrosis (FIB)‐4 index, a non‐invasive index of hepatic fibrosis, was calculated using the following formula: age (years) × aspartate aminotransferase (AST) (U/L)/(platelet count [10^9^/l] × (alanine aminotransferase (ALT) [U/L])^1/2^).

### 2.4 Statistical analysis

SPSS Statistics 27.0 (IBM, Inc.) was used for all the statistical analyses. Data are expressed as mean ± standard error. The relationships between the immunostaining groups and the prevalence of each disease were evaluated using Pearson's chi‐squared test. The relationship of each pathophysiological factor with immunostaining was evaluated by ANOVA. In addition, factors that contributed to the dense nuclear staining of NRF2 were identified by univariate and multivariate analyses. *p*‐Values of <0.05 indicated statistical significance.

## 3 RESULTS

### 3.1 Characteristics of patients in each disease group

Table 1 details the characteristics of the study patients from which 50 HCCs developed from NASH, 49 HCCs developed from CHc, and 48 liver metastases of CRC were analyzed. They were 68.2 ± 0.8 years old and 75.5% (111/147) were men. Patients with CRC were younger than those with NASH or CHc. Patients with NASH had higher a BMI and prevalence of HT and DM than those with CHc and CRC. Regarding blood biochemistry, platelet counts in patients with NASH tended to be lower; however, this was not significantly different compared with those with CRC. Furthermore, platelet counts in patients with CHc were lower than in those with NASH and CRC. Prothrombin time (PT%) was lower in patients with NASH and CHc. In parallel with these changes in hepatic spare ability, FIB‐4 index was higher in patients with CHc than in those with NASH and CC. ICG15 in patients with CHc was higher than in those with CRC; however, it was not significantly different compared with those with NASH. Serum AST was higher in patients with CHc than in those with CRC, while serum ALT was higher in patients with NASH compared with those with CRC. Serum gamma‐glutamyl transferase (γ‐GT) and alkaline phosphatase (ALP) in patients with NASH and CRC tended to be higher compared with those with CHc; however, this was not significant.

**TABLE 1:** Characteristics of 147 patients including 50 NASH with HCC, 49 CHc with HCC, and 48 liver metastasis of CRC.

Lifestyle‐related disease definitions and clinical laboratory measurements						
	CRC (group 1) (*n* = 48)	NASH (group 2) (*n* = 50)	CHc (group 3) (*n* = 49)	*p*‐Value		
				1 vs. 2	1 vs. 3	2 vs. 3
Age (years)	64.5 ± 1.9	70.5 ± 1.4	69.5 ± 1.1	0.014	0.053	0.876
Sex, male/female (*n*)	32/16	40/10	39/10	0.135	0.151	0.960
BMI	22.4 ± 0.5	25.0 ± 0.6	21.9 ± 0.4	0.001	0.781	<0.001
Lifestyle‐related diseases						
Hypertension (%)	17.9	88.0	67.3	<0.001	0.053	0.013
Diabetes mellitus (%)	39.6	74.0	51.0	<0.001	0.258	0.018
Dyslipidemia (%)	39.6	58.0	22.4	0.068	0.068	<0.001
Hepatic abnormalities						
Platelet, ×10^10^/L	19.9 ± 0.6	17.8 ± 0.7	13.7 ± 0.7	0.096	<0.001	<0.001
PT, %	95.8 ± 1.0	86.1 ± 1.8	82.5 ± 2.2	<0.001	<0.001	0.325
Albumin, g/dL	4.2 ± 0.1	4.1 ± 0.1	3.9 ± 0.1	0.391	0.016	0.293
AST, U/L	30.1 ± 2.0	43.6 ± 4.3	46.4 ± 5.4	0.061	0.018	0.877
ALT, U/L	26.4 ± 2.5	58.2 ± 12.9	44.9 ± 6.9	0.029	0.310	0.506
ALP, U/L	323.0 ± 23.6	310.8 ± 21.0	300.2 ± 16.0	0.907	0.712	0.927
γ‐GT, U/L	95.5 ± 16.9	95.0 ± 11.4	62.8 ± 8.9	1.000	0.169	0.169
FIB‐4 index	2.5 ± 0.2	4.6 ± 0.5	8.1 ± 1.1	0.079	<0.001	0.001
ICG15, %	10.6 ± 0.8	12.4 ± 1.2	14.7 ± 1.1	0.448	0.017	0.251
Glucose and lipid profiles						
FPG, mg/dL	108.3 ± 3.5	135.6 ± 8.6	131.1 ± 10.9	0.033	0.119	0.918
HbA1c, %	6.0 ± 0.2	6.6 ± 0.1	6.2 ± 0.2	0.020	0.775	0.105
HDL‐C, mg/dL	60.9 ± 2.3	50.1 ± 2.2	53.1 ± 2.7	0.004	0.064	0.672
LDL‐C, mg/dL	124.2 ± 6.1	101.9 ± 4.2	96.3 ± 4.6	0.005	<0.001	0.729
TG, mg/dL	131.3 ± 10.1	139.1 ± 12.3	102.1 ± 7.5	0.852	0.121	0.034
Characteristics of HCCs						
Differentiation (well/moderately/poorly)		1/41/8	1/34/14			0.320
Maximum size, cm		49.4 ± 4.2	34.2 ± 2.6			0.003
Number of HCCs		1.4 ± 0.2	1.6 ± 0.1			0.631
Tumor marker						
AFP, mg/dL	4.1 ± 0.5	1769.4 ± 1213.4	1783.6 ± 1204.8	0.857	0.855	1.000
PIVKAII, mg/dL	22.4 ± 3.6	4824.8 ± 2104.7	3732.5 ± 1685.1	0.634	0.762	0.909
CEA, mg/dL	57.7 ± 21.8	2.7 ± 0.3	3.7 ± 0.6	0.101	0.097	0.999
CA19‐9, mg/dL	105.1 ± 40.7	19.0 ± 2.5	33.2 ± 6.6	0.216	0.376	0.971

Histological scoring of NASH by SAF and CHc by the New Inuyama classification in non‐tumorous areas						
SAF score						
Steatosis (0–3)	0.4 ± 0.1	0.9 ± 0.1		<0.001		
Activity (perilobular inflammation, 0–2)	0.2 ± 0.1	1.5 ± 0.1		<0.001		
Activity (ballooning, 0–2)	0.2 ± 0.1	1.9 ± 0.0		<0.001		
Activity (total, 0–4)	0.4 ± 0.1	3.5 ± 0.1		<0.001		
Fibrosis (0–4)	0.1 ± 0.1	3.2 ± 0.2		<0.001		
New Inuyama classification						
Activity (0–3)	0.2 ± 0.1		2.0 ± 0.9		<0.001	
Fibrosis (0–4)	0.2 ± 0.1		3.7 ± 0.1		<0.001	

*Note*: Values are presented as the group means ± standard error. To compare between two groups, all dependent variables were analyzed by ANOVA. Categorical variables were analyzed using Pearson's chi‐squared test.

Abbreviations: AFP, alpha fetoprotein; ALP, alkaline phosphatase; ALT, alanine aminotransferase; AST, aspartate aminotransferase; BMI, body mass index; CA19‐9, carbohydrate antigen 19‐9; CEA, carcinoembryonic antigen; CHc, chronic viral hepatitis C; CHc, chronic viral hepatitis C; CRC, colorectal cancer; CRC, colorectal cancer; FPG, fasting plasma glucose; HbA1c, hemoglobin A1c; HCC, hepatocellular carcinoma; HDL‐C, high‐density lipoprotein‐cholesterol; ICG, indocyanine green clearance test; LDL‐C, low‐density lipoprotein‐cholesterol; NASH, non‐alcoholic steatohepatitis; PIVKA‐II, protein induced by vitamin K absence II; PT, prothrombin time; SAF, steatosis, activity, and fibrosis; SAF, steatosis, activity, and fibrosis; TG, triglyceride; γ‐GT, gamma‐glutamyl transferase.

Patients with NASH had worse glucose tolerance, FPG, and HbA1c than those with CRC. Patients with CRC had higher serum HDL‐C and LDL‐C than those with NASH and CHc, while patients with CHc had lower serum TG than those with NASH and CRC. However, the data might not reflect the severity of DM and DL because they combined the information of treated and untreated patients.

In a histopathological analysis by SAF score, patients with NASH had higher grades of steatosis, activity, and fibrosis than those with CRC (Table 1, lower panel). Furthermore, in the histopathological analysis by New Inuyama classification, patients with CHc had higher grades of activity and fibrosis than those with CRC (Table  1, lower panel).

In the pathophysiological characteristics of HCC, as shown in Table 1 (upper panel), patients with NASH had a higher maximum size of HCC, as previously reported.^23^ Patients with NASH and CHc had a higher tendency to express tumor markers of HCC, including serum AFP and PIVKA‐II, compared with those with CRC, while patients with CRC had a higher tendency to express tumor markers of CRC, including serum CEA and CA19‐9, compared with those with NASH and CHc; however, there was no significance because of the large standard errors.

### 3.2 Immunohistochemical analyses of NRF2 and KEAP1 in each disease group

NRF2 was mainly stained and expressed in cytosol of hepatocytes in both non‐tumorous and tumorous areas (Figure 1), and NRF2 was stained in some small inflammatory cells that had infiltrated the liver; however, this was minor. Meanwhile, some hepatocytes in non‐tumorous areas and some HCCs had dense immunostaining of NRF2 in the nucleus (Figure 1), which is considered to indicate NRF2 translocation into the nucleus.

Table 2 shows the results of immunohistochemical analyses of NRF2 and KEAP1 in the patients of each disease group. In non‐tumorous areas of the liver, the CRC group had higher NRF2 intensity compared with those with NASH and CHc; however, there was no significance between NASH and CHc. Furthermore, the NASH and CHc groups had more patients that had nuclear staining of NRF2 compared with CRC. Moreover, the CHc group exhibited the highest number of nuclei that was densely stained by NRF2, followed by the NASH group and the CRC group. Patients in the CRC group had higher KEAP1 intensity compared with those in the NASH and CHc groups; however, there was no significant difference between NASH and CHc. In tumorous areas of NASH and CHc, namely HCC, NRF2 intensity and localization and KEAP1 intensity did not differ significantly between the NASH and CHc groups.

**TABLE 2:** Immunohistochemical analyses of NRF2 and KEAP1 in each disease type.

	CRC (group 1) (*n* = 48)	NASH (group 2) (*n* = 50)	CHc (group 3) (*n* = 49)	*p*‐Value		
				1 vs. 2	1 vs. 3	2 vs. 3
Non‐tumorous areas						
NRF2 intensity (low/medium/high)	5/33/10	15/32/3	17/30/2	0.013	0.002	0.827
NRF2 localization (cytosol/+ nucleus)	26/22	7/43	1/48	<0.001	<0.001	0.029
Dense NRF2 staining in nucleus (*n*)	2.4 ± 0.3	4.7 ± 0.4	8.6 ± 0.5	0.001	<0.001	<0.001
KEAP1 intensity (low/high)	33/15	44/6	47/2	0.020	<0.001	0.148
Tumorous areas						
NRF2 intensity (low/medium/high)		26/22/2	23/24/2			0.878
NRF2 localization (cytosol/+ nucleus)		0/50	0/49			
Dense NRF2 staining in nucleus (low/medium/high)		27/13/10	35/7/7			0.187
KEAP1 intensity (low/high)		42/8	43/6			0.592

*Note*: Values are presented as the group mean ± standard error. To compare between two groups, all dependent variables were analyzed by ANOVA. Categorical variables were analyzed using Pearson's chi‐squared test.

Abbreviations: CRC, colorectal cancer; KEAP1, Kelch‐like Ech‐associated protein; NRF2, nuclear factor E2‐related factor 2.

### 3.3 Comparison of patient information with NRF2 intensity in non‐tumorous areas

Table 3 (upper panel) shows comparisons of patients with low‐, medium‐, and high‐intensity NRF2 immunostaining in non‐tumorous areas of the liver. Age, sex, BMI, and DM prevalence did not differ significantly according to NRF2 immunostaining. The prevalence of HT and DL was lower in the high‐intensity NRF2 group compared with the low‐intensity NRF2 group; however, the lipid profiles did not differ significantly. Hepatic abnormalities including liver spare ability, liver injury, and FIB‐4 index also did not differ significantly between the expression groups. Likewise, glucose metabolism did not differ significantly. Furthermore, hepatic inflammation and fibrosis assessed by SAF score between CRC and NASH (total *n* = 98) were more severe in the low‐intensity NRF2 group than in the high‐intensity NRF2 group (Table 3, middle panel). Similarly, hepatic inflammation and fibrosis assessed by the New Inuyama classification between CRC and CHc (total *n* = 97) were more severe in the low‐intensity NRF2 group than in the high‐intensity NRF2 group (Table 3, lower panel).

**TABLE 3:** Comparison of NRF2 immunostaining in non‐tumorous areas of 147 patients.

Lifestyle‐related disease definitions and clinical laboratory measurements						
CRC & NASH & CHc	Low (*n* = 37)	Medium (*n* = 95)	High (*n* = 15)	*p*‐Value		
				Low vs. medium	Low vs. high	Medium vs. high
Age, years	69.8 ± 1.4	67.3 ± 1.2	70.1 ± 2.7	0.438	0.997	0.620
Sex, male/female (*n*)	25/12	73/22	13/2	0.274	0.160	0.392
BMI	23.0 ± 0.5	23.1 ± 0.4	23.0 ± 1.1	0.991	0.999	0.990
Lifestyle‐related diseases						
Hypertension (%)	78.4	68.4	40.0	0.256	0.008	0.032
Diabetes mellitus (%)	59.5	54.7	46.7	0.623	0.400	0.380
Dyslipidemia (%)	51.4	38.9	20.0	0.195	0.038	0.156
Hepatic abnormalities						
Platelets, ×10^10^/L	15.9 ± 0.9	17.6 ± 0.6	16.9 ± 1.1	0.251	0.847	0.867
PT, %	87.8 ± 1.8	87.8 ± 1.5	90.4 ± 2.4	1.000	0.805	0.769
ICG15, %	13.7 ± 1.2	12.1 ± 0.8	12.6 ± 1.7	0.529	0.885	0.970
Albumin, g/dL	4.1 ± 0.1	4.1 ± 0.1	4.1 ± 0.1	0.795	0.993	0.943
AST, U/L	39.4 ± 3.9	39.0 ± 3.3	48.9 ± 7.9	0.998	0.557	0.465
ALT, U/L	43.8 ± 10.9	41.7 ± 6.5	51.3 ± 10.9	0.984	0.919	0.846
γ‐GT, U/L	83.6 ± 14.3	78.7 ± 8.1	121.6 ± 36.8	0.957	0.345	0.196
ALP, U/L	270.0 ± 19.2	319.5 ± 15.4	361.0 ± 33.9	0.167	0.090	0.538
FIB‐4 index	5.3 ± 0.8	4.8 ± 0.5	6.2 ± 1.8	0.881	0.862	0.637
Glucose metabolism and lipid profiles						
FPG, mg/dL	137.0 ± 11.2	122.4 ± 5.5	108.2 ± 7.8	0.379	0.230	0.644
HbA1c, %	6.4 ± 0.2	6.2 ± 0.1	6.2 ± 0.3	0.616	0.731	0.979
HDL‐C, mg/dL	52.3 ± 3.6	55.0 ± 1.7	58.8 ± 3.4	0.722	0.470	0.723
LDL‐C, mg/dL	109.0 ± 6.7	106.9 ± 4.0	115.7 ± 5.7	0.959	0.844	0.692
TG, mg/dL	132.9 ± 10.5	122.9 ± 8.2	116.6 ± 12.6	0.766	0.760	0.951

Histological scoring by SAF						
CRC & NASH	Low (*n* = 20)	Medium (*n* = 65)	High (*n* = 13)	*p*‐Value		
				Low vs. medium	Low vs. high	Medium vs. high
SAF score						
Steatosis (0–3)	1.0 ± 0.1	0.6 ± 0.1	0.5 ± 0.2	0.074	0.058	0.670
Activity (perilobular inflammation, 0–2)	1.2 ± 0.2	0.8 ± 0.1	0.6 ± 0.2	0.199	0.106	0.610
Activity (ballooning, 0–2)	1.6 ± 0.2	1.0 ± 0.1	0.5 ± 0.2	0.083	0.003	0.093
Activity (total 0–4)	2.8 ± 0.3	1.9 ± 0.2	1.1 ± 0.4	0.105	0.014	0.232
Fibrosis (0–4)	2.4 ± 0.4	1.6 ± 0.2	0.8 ± 0.4	0.183	0.035	0.312

New Inuyama classification						
CRC & CHc	Low (*n* = 22)	Medium (*n* = 63)	High (*n* = 12)	*p*‐Value		
				Low vs. medium	Low vs. high	Medium vs. high
New Inuyama classification						
Activity (0–3)	1.5 ± 0.2	1.1 ± 0.1	0.6 ± 0.3	0.154	0.031	0.314
Fibrosis (0–4)	2.7 ± 0.4	1.9 ± 0.2	0.8 ± 0.4	0.178	0.011	0.129

*Note*: Values are presented as the group mean ± standard error. To compare between two groups, all dependent variables were analyzed by ANOVA. Categorical variables were analyzed using Pearson's chi‐squared test.

Abbreviations: CA19‐9, carbohydrate antigen 19‐9; CEA, carcinoembryonic antigen; CHc, chronic viral hepatitis C; CRC, colorectal cancer; FPG, fasting plasma glucose; HbA1c, hemoglobin A1c; HDL‐C, high‐density lipoprotein‐cholesterol; ICG, indocyanine green clearance test; LDL‐C, low‐density lipoprotein‐cholesterol; NRF2, nuclear factor E2‐related factor 2; PIVKA‐II, protein induced by vitamin K absence II; SAF, steatosis, activity, and fibrosis; TG, triglyceride; AFP, alpha fetoprotein; γ‐GT, gamma‐glutamyl transferase.

### 3.4 Comparison of patient information according to NRF2 localization in non‐tumorous areas

Table 4 (upper panel) shows comparisons of patients with NRF2 localization in the cytosol or the cytosol and/or nucleus (+ nucleus group) of hepatocytes in non‐tumorous areas. Age, sex, BMI, and prevalence of lifestyle‐related diseases did not significantly differ between the two groups according to NRF2 localization. Interestingly, platelet count and PT%, which reflect liver spare ability and liver fibrosis in hepatic abnormalities, were significantly lower in the + nucleus group than in the cytosol group. Moreover, FIB‐4 index was also significantly higher in the + nucleus group than in the cytosol group. Meanwhile, γ‐GT was lower in the + nucleus group compared with the cytosol group; however, other biomarkers of hepatic abnormalities did not differ between the two groups. Likewise, glucose metabolism and lipid profiles did not significantly differ between the two groups. Histopathological hepatic steatosis, inflammation and fibrosis assessed by SAF score were compared between CRC and NASH (total *n* = 98) and were found to be significantly more severe in the + nucleus group than in the cytosol group (Table 4, middle panel). Moreover, histopathological hepatic inflammation and fibrosis assessed by the New Inuyama classification and compared in CRC and CHc (total *n* = 97) were significantly more severe in the + nucleus group than in the cytosol group (Table 4, lower panel).

**TABLE 4:** Comparison of NRF2 localization in non‐tumorous areas in 147 patients.

Lifestyle‐related disease definitions and clinical laboratory measurements			
CRC & NASH & CHc	Cytosol (*n* = 34)	+ Nucleus (*n* = 113)	*p*‐Value
Age, years	66.5 ± 2.0	68.7 ± 1.0	0.303
Sex, male/female (*n*)	24/10	87/26	0.447
BMI	23.0 ± 0.8	23.1 ± 0.3	0.849
Lifestyle‐related, diseases			
Hypertension (%)	58.8	70.8	0.189
Diabetes mellitus (%)	50.0	56.6	0.495
Dyslipidemia (%)	38.2	40.7	0.796
Hepatic abnormalities			
Platelets, ×10^10^/L	19.2 ± 0.8	16.5 ± 0.5	0.013
PT, %	92.7 ± 1.6	86.6 ± 1.3	0.021
ICG15, %	10.9 ± 0.9	13.1 ± 0.8	0.073
Albumin, g/dL	4.2 ± 0.1	4.0 ± 0.1	0.053
AST, U/L	34.6 ± 2.9	41.8 ± 3.1	0.221
ALT, U/L	27.8 ± 3.1	47.9 ± 6.5	0.098
γ‐GT, U/L	122.3 ± 23.5	72.8 ± 6.1	0.004
ALP, U/L	327.5 ± 22.6	306.4 ± 13.7	0.428
FIB‐4 index	3.5 ± 0.6	5.6 ± 0.5	0.012
Glucose metabolism and lipid profiles			
FPG, mg/dL	108.3 ± 5.2	129.0 ± 5.7	0.069
HbA1c, %	6.0 ± 0.1	6.4 ± 0.1	0.089
HDL‐C, mg/dL	59.6 ± 3.1	53.5 ± 1.6	0.092
LDL‐C, mg/dL	123.4 ± 8.5	104.1 ± 3.2	0.041
TG, mg/dL	126.7 ± 10.5	124.3 ± 7.2	0.852

Histological scoring by SAF			
CRC & NASH	Cytosol (*n* = 33)	+ Nucleus (*n* = 65)	*p*‐Value
SAF score			
Steatosis (0–3)	0.5 ± 0.1	0.8 ± 0.1	0.007
Activity (perilobular inflammation, 0–2)	0.5 ± 0.1	1.1 ± 0.1	<0.001
Activity (ballooning, 0–2)	0.4 ± 0.1	1.4 ± 0.1	<0.001
Activity (total, 0–4)	0.9 ± 0.2	2.5 ± 0.2	<0.001
Fibrosis (0–4)	0.5 ± 0.2	2.3 ± 0.2	<0.001

New Inuyama classification			
CRC & CHc	Cytosol (*n* = 27)	+ Nucleus (*n* = 70)	*p*‐Value
New Inuyama classification			
Activity (0–3)	0.2 ± 0.1	1.5 ± 0.1	<0.001
Fibrosis (0–4)	0.2 ± 0.1	2.6 ± 0.2	<0.001

*Note*: Values are presented as the group mean ± standard error. To compare between two groups, all dependent variables were analyzed by ANOVA. Categorical variables were analyzed Lining Pearson's chi‐squared test.

Abbreviations: ALP, alkaline phosphatase; ALT, alanine aminotransferase; AST, aspartate aminotransferase; BMI, body mass index; CHc, chronic viral hepatitis C; CRC, colorectal cancer; FPG, fasting plasma glucose; HbA1c, hemoglobin A1c; HDL‐C, high‐density lipoprotein‐cholesterol; ICG, indocyanine green clearance test; LDL‐C, low‐density lipoprotein‐cholesterol; NRF2, nuclear factor E2‐related factor 2; PT, prothrombin time; SAF, steatosis, activity, and fibrosis.TG, triglyceride; γ‐GT, gamma‐glutamyl transferase.

To clarify whether nuclear localization of NRF2, which can indicate NRF2 translocation into the nucleus, correlated with liver pathophysiology, the number of densely NRF2‐immunostained nuclei of non‐tumor hepatocytes was analyzed and compared with patient information and histopathology. Interestingly, the number of densely NRF2‐immunostained nuclei had an opposite correlation with hepatic spare ability such as platelet counts and PT% (Figure 2A), mirroring the results in Table 4 (upper panel). Moreover, the number of densely NRF2‐immunostained nuclei had a positive correlation with liver inflammation and fibrosis assessed by SAF score in CRC and NASH and the New Inuyama classification in CRC and CHc (Figure 2B and 2C). However, there was no correlation with liver injury markers such as AST, ALT, γ‐GT, and ALP (Figure 2A).

These results suggest that the localized immunostaining of NRF2 in the nuclei of non‐tumorous hepatocytes, namely, the translocation of NRF2 into the nucleus, is associated with liver inflammation and fibrosis.

### 3.5 Evaluation of HCC by intensity and localization of NRF2 in tumorous areas

Table S1 shows comparisons of patients with low‐, medium‐, and high‐intensity NRF2 immunostaining in HCC. Characteristics of HCC did not differ significantly according to the intensity of NRF2 immunostaining.

Likewise, these did not differ according to the density of NRF2 in the nucleus in HCC (Table S1). In the present study, follow‐up of patient prognosis was not performed.

### 3.6 Comparison of patient information according to KEAP1 intensity in non‐tumorous and HCC areas

KEAP1 was uniformly stained in the cytosol of hepatocytes both in non‐tumorous areas and HCC areas (Figure 1). Table 5 (upper panel) presents comparisons of patients with low and high KEAP1 immunostaining intensity in non‐tumorous areas. In examinations of blood biochemistry, PT% and albumin were lower and FIB‐4 index was higher in the low‐intensity group than in the high‐intensity group. Moreover, hepatic inflammation assessed by SAF score in CRC and NASH were more severe in the low‐intensity KEAP1 group than in the high‐intensity KEAP1 group (Table 5, middle panel). In addition, hepatic inflammation and fibrosis assessed by the New Inuyama classification in CRC and CHc were more severe in the low‐intensity KEAP1 group compared with those in the high‐intensity KEAP1 group (Table 5, lower panel).

**TABLE 5:** Comparison of KEAP1 immunostaining in non‐tumorous areas of 147 patients.

Lifestyle‐related disease definitions and clinical laboratory measurements			
CRC & NASH & CHc	Low (*n* = 124)	High (*n* = 23)	*p*‐Value
			Low vs. high
Age, years	68.2 ± 0.9	68.3 ± 3.0	0.961
Sex, male/female (*n*)	95/29	16/7	0.470
BMI	23.2 ± 0.4	12.5 ± 0.6	0.373
Lifestyle‐related diseases			
Hypertension (%)	68.5	65.2	0.753
Diabetes mellitus (%)	55.6	52.2	0.759
Dyslipidemia (%)	40.3	39.1	0.915
Hepatic abnormalities			
Platelets, ×10^10^/L	16.9 ± 0.5	18.2 ± 1.0	0.255
PT, %	87.2 ± 1.3	92.4 ± 1.8	0.026
ICG15, %	12.7 ± 0.6	11.7 ± 1.9	0.625
Albumin, g/dL	4.0 ± 0.0	4.3 ± 0.1	0.003
AST, U/L	41.5 ± 2.8	32.6 ± 5.0	0.126
ALT, U/L	43.3 ± 5.2	42.9 ± 16.8	0.981
γ‐GT, U/L	85.4 ± 7.6	78.5 ± 24.6	0.792
ALP, U/L	314.2 ± 12.1	295.7 ± 36.9	0.637
FIB‐4 index	5.4 ± 0.5	3.4 ± 0.6	0.010
Glucose metabolism and lipid profiles			
FPG, mg/dL	126.2 ± 5.5	117.0 ± 6.4	0.278
HbA1c, %	6.3 ± 0.1	6.1 ± 0.3	0.577
HDL‐C, mg/dL	53.4 ± 1.6	60.4 ± 3.0	0.051
LDL‐C, mg/dL	108.7 ± 3.6	106.3 ± 6.7	0.755
TG, mg/dL	128.0 ± 7.2	110.2 ± 8.2	0.108

Histological scoring by SAF			
CRC & NASH	Low (*n* = 77)	High (*n* = 21)	*p*‐Value
			Low vs. high
SAF score			
Steatosis (0–3)	0.7 ± 0.1	0.6 ± 0.2	0.647
Activity (perilobular inflammation, 0–2)	1.0 ± 0.1	0.6 ± 0.2	0.039
Activity (ballooning, 0–2)	1.2 ± 0.1	0.7 ± 0.2	0.051
Activity (total, 0–4)	2.1 ± 0.2	1.3 ± 0.4	0.040
Fibrosis (0–4)	1.8 ± 0.2	1.1 ± 0.3	0.075

New Inuyama classification			
CRC & CHc	Low (*n* = 80)	High (*n* = 17)	*p*‐Value
			Low vs. high
New Inuyama classification			
Activity (0–3)	1.3 ± 0.1	0.5 ± 0.2	0.002
Fibrosis (0–4)	2.2 ± 0.2	0.8 ± 0.4	0.003

*Note*: Values are presented as the group mean ± standard error. To compare between two groups, all dependent variables were analyzed by ANOVA. Categorical variables were analyzed using Pearson's chi‐squared test.

Abbreviations: ALP, alkaline phosphatase; ALT, alanine aminotransferase; AST, aspartate aminotransferase; BMI, body mass index; CHc, chronic viral hepatitis C; CRC, colorectal cancer; FPG, fasting plasma glucose; HbA1c, hemoglobin A1c; HDL‐C, high‐density lipoprotein‐cholesterol; ICG, indocyanine green clearance test; KEAP1, Kelch‐like Ech‐associated protein; LDL‐C, low‐density lipoprotein‐cholesterol; PT, prothrombin time; SAF, steatosis, activity, and fibrosis; TG, triglyceride; γ‐GT, gamma‐glutamyl transferase.

KEAP1 and NRF2 expression in non‐tumorous areas (Figure S1) and HCC areas (Figure S1) was compared to clarify whether KEAP1 could inhibit and/or regulate NRF2. KEAP1 intensity did not correlate with the intensity, localization, or nuclear staining intensity of NRF2 in either non‐tumorous areas (Figure S1) or HCC areas (Figure S1).

Table S2 shows comparisons of patients with low and high KEAP1 immunostaining intensity in HCC areas. HCC characteristics including maximum tumor size, tumor number, histopathological differentiation, T factor, and tumor markers including AFP and PIVKA‐II did not differ significantly between the two groups according to KEAP1 immunostaining intensity.

## 4 DISCUSSION

In the present study, we demonstrated that NRF2 expression was correlated with the pathological conditions of chronic liver diseases such as NASH and CHc. In detail, weakened immunostaining intensity in cytosol of hepatocytes and nuclear translocation of NRF2 correlated with inflammation and fibrosis in the liver. Although NRF2 localization and dense nuclear NRF2 staining, namely, translocation of NRF2 into nucleus in non‐tumorous areas, were more frequently observed in the CHc group compared with NASH (Table 2), the correlation was common in the NASH and CHc groups. These results indicate that NRF2 was accumulated in the nucleus and activated by oxidative stress in the NASH and CHc groups.

NRF2 is activated by translocation to the nucleus and acts to protect cells. A previous study reported that NRF2 transcription factor activity was increased in patients with lobular inflammation of chronic liver disease by RNA‐seq analysis.^24^ However, the immunohistochemical behavior of NRF2 in chronic liver disease had not been made fully clear, probably because the analyses were limited by the small number of patients. Moreover, a small number of patients with advanced liver fibrosis or cirrhosis was included in the study,^24^ and a correlation between NRF2 expression level and liver fibrosis was not demonstrated. In this study, we newly demonstrated that patients with NRF2 nuclear localization and dense nuclear NRF2 staining in non‐tumorous areas had more severe hepatic inflammation and fibrosis (Table 4; Figure 2). The nuclear translocation of NRF2 in non‐tumorous areas was a common finding in chronic hepatitis of NASH and CHc, while hepatic fibrosis in CHc was more severe than that in NASH, as demonstrated by decreased hepatic spare ability features such as platelet counts and PT (Table 1, upper panel). These results may reflect a larger malignant potential in CHc compared with NASH, as evidenced by carcinogenesis occurring at a younger age in patients with CHc compared with those with NASH^25^ because hepatic fibrosis is a major risk factor for carcinogenesis. Thus, the immunohistochemical expression of NRF2 may be related not only to the progression of chronic liver disease but also hepatic carcinogenesis.

NRF2, which is a master regulator of the cellular adaptive response to oxidative stress, was reported to have a protective role in the gene regulatory program of the antioxidant response against liver diseases including NASH in animal models.^18^ *NRF2* deletion leads to the development of inflammation and fibrosis in the liver with nutritional steatohepatitis caused by impaired antioxidative stress including glutathione, catalase, and superoxide dismutase regulated by NRF2.^26^ Especially, NRF2 expressed in hepatocytes was demonstrated to have important roles in experiments using genetically engineered mice with hepatocyte‐specific *Nrf2* deletion and activation.^24^, ^27^ Furthermore, it has been reported that NRF2 can regulate inflammation independently of the antioxidant response through direct binding of pro‐inflammatory promotor regions in macrophages^28^; thus, NRF2 may contribute to the suppression of disease progression by regulating immune cells, even in an oxidative stress‐independent manner in non‐tumorous areas in the present study. Together with previous reports and our recent data in animal studies, these results suggested that NRF2 could have anti‐inflammatory and anti‐fibrotic roles in inhibiting the development of chronic liver diseases.

Despite playing a protective role in inflammatory disease, constitutive activation of NRF2 in cancer cells including HCC and gallbladder carcinoma was reported to contribute to chemo‐ and radioresistance of cancer.^19^, ^20^ In addition, elevated expression levels of NRF2 in HCC have been reported to be associated with overall and progression‐free survival.^29^ In the present study, HCC exhibited dense nuclear immunostaining of NRF2, consistent with previous reports; however, the expression level and nuclear staining density of NRF2 were not associated with the degree of differentiation and pathological stage of HCC (Table S1).

Loss of KEAP1 activity as a result of somatic mutations has been reported in HCC,^14^, ^15^, ^19^ implying constitutive activation of NRF2. In the present study, KEAP1 expression levels had an opposite correlation with liver inflammation and fibrosis in non‐tumorous areas (Table 5), while KEAP1 expression was not associated with HCC progression and pathophysiology (Table S2). Although KEAP1 expression was not correlated with NRF2 expression and nuclear staining in both tumorous and non‐tumorous areas (Figure S1), low‐intensity KEAP1 immunostaining tended to be more prevalent in patients with nuclear translocation of NRF2 than in those with high‐intensity KEAP1 immunostaining in non‐tumorous areas (Figure S1), as demonstrated in *Keap1* knockdown mice, in which downregulation of KEAP1 activated NRF2 constitutively.^18^ These results suggest that low expression of KEAP1 induces nuclear translocation of NRF2; however, the link was not observed in HCC areas (Figure S1).

The development of fibrosis in chronic liver disease induces hepatocarcinogenesis, and its control is an important issue in the treatment of liver disease.^30^, ^31^ The frequency of hepatocarcinogenesis is high in obese, diabetic, heavy alcohol drinkers, and older patients^4^, ^32^; moreover, a strong correlation has been shown between the severity of NASH and the degree of oxidative stress.^33^ In CHc, excessive iron deposition induces oxidative stress and leads to inflammation and fibrosis progression in the liver.^34^ ROS induces hepatocyte death, inflammatory responses by macrophages, and activation of hepatic stellate cells, leading to the development of liver fibrosis^35^, ^36^; hence, the control/suppression of oxidative stress including the generation of ROS is important in chronic liver disease.

In this study, NRF2 staining intensity and nuclear translocation were correlated with hepatic functional capacity, inflammation, and fibrosis. In the absence of drugs that inhibit oxidative stress, inflammation, and fibrosis in chronic liver disease, the role of NRF2 as a comprehensive protective factor may provide a new therapeutic option for the prevention and treatment of hepatic inflammation and fibrosis.

The present study has several limitations. First, the study patients underwent liver resection surgery at a single center in an Asian country and human liver materials with not only tumorous but also non‐tumorous areas developed from NASH and CHc were examined. Moreover, the amount of control liver with CRC was quite large.

Second, it was difficult to completely evaluate the function and mechanism of NRF2 in the development and carcinogenesis of chronic liver disease because this was a cross‐sectional study. Furthermore, it was hard to evaluate whether NRF2 expression correlated with the prognosis of cirrhosis and HCC because follow‐up of the patients could not be checked.

In conclusion, the weakened immunostaining intensity and nuclear translocation of NRF2 were correlated with pathological conditions of liver inflammation and fibrosis in chronic liver disease. Taken together with the present data and previous studies, these results suggest that NRF2 might have a protective role against the development and carcinogenesis of chronic liver disease including NASH and CHc in humans.

## AUTHOR CONTRIBUTIONS

**Keii To:** Conceptualization (lead); data curation (lead); formal analysis (lead); investigation (lead); writing – original draft (lead); writing – review and editing (lead). **Kosuke Okada:** Conceptualization (lead); data curation (lead); formal analysis (lead); funding acquisition (lead); methodology (lead); project administration (lead); writing – original draft (lead); writing – review and editing (lead). **Takahisa Watahiki:** Data curation (lead); formal analysis (lead); investigation (equal); writing – review and editing (equal). **Hideo Suzuki:** Writing – review and editing (equal). **Kiichiro Tsuchiya:** Supervision (lead); writing – review and editing (equal). **Katsutoshi Tokushige:** Supervision (lead); writing – review and editing (equal). **Masakazu Yamamoto:** Data curation (lead); resources (lead); writing – review and editing (equal). **Shun‐ichi Ariizumi:** Conceptualization (equal); data curation (lead); project administration (equal); resources (lead); writing – review and editing (equal). **Junichi Shoda:** Conceptualization (equal); formal analysis (equal); funding acquisition (equal); writing – review and editing (equal).

## FUNDING INFORMATION

This work was supported by the Japan Society for the Promotion of Science (JSPS) KAKENHI (Grant numbers JP20H04119, JP21K07933, JP21H03372, JP21H03010, and JP22K08072) and by the Japanese Society of Gastroenterology (JSGE) Grant.

## CONFLICT OF INTEREST STATEMENT

The authors declare that they have no conflicts of interest.

## ETHICS STATEMENT

We conducted this study in accordance with the Ethical Guidelines for Medical and Health Research Involving Human Subjects, published by the Ministry of Education, Culture, Sports, Science, and Technology and the Ministry of Health, Labour, and Welfare of Japan. The study was approved by the ethics committee of the University of Tsukuba Hospital (approval No. R01‐147) and Tokyo Women's Medical University (approval No. 4932) conforms to the provisions of the Declaration of Helsinki. This study was registered with the University Hospital Medical Information Network (UMIN) Clinical Trial Registry (UMIN ID 000037080). Approval of the research protocol: the ethics committee of the University of Tsukuba Hospital (approval No. R01‐147) and Tokyo Women's Medical University (approval No. 4932). Registry and the Registration No. of the study/trial: UMIN ID 000037080.

## CONSENT

Written informed consent was obtained from all patients.

## Supporting information


Figure S1:

Table S1:

Table S2:

## Data Availability

The datasets are available from the corresponding author on reasonable request.
