# Supplementary figures and images for: Immunohistochemical expression of NRF2 is correlated with the magnitude of inflammation and fibrosis in chronic liver disease
Source: Cancer Med. 2023 Sep 21;12(19):19423–37. doi: 10.1002/cam4.6538 (PMC10587934; doi:10.1002/cam4.6538)

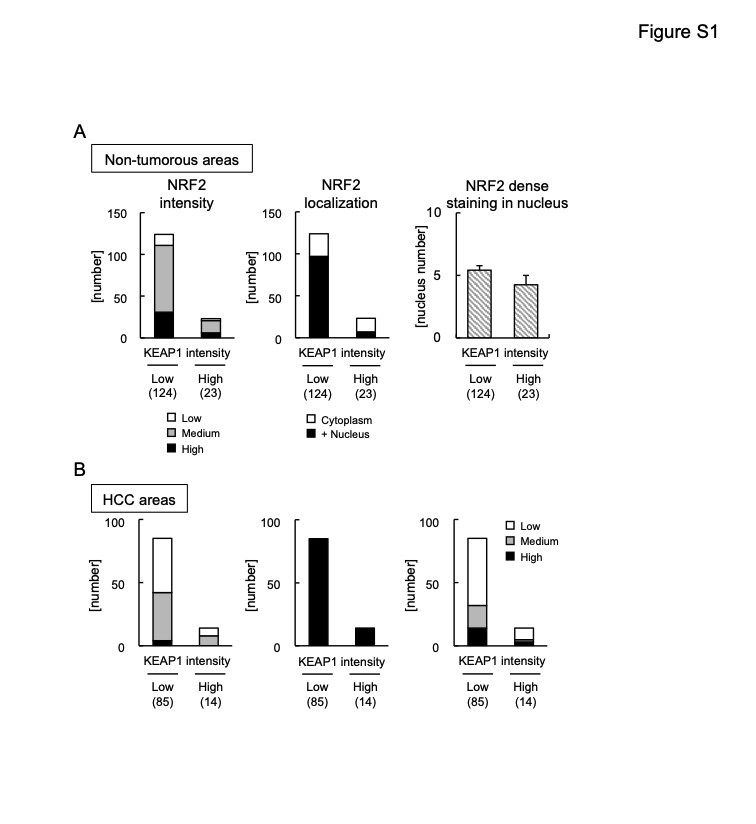

Supplement: Supplementary file 1 — Figure S1: [file CAM4-12-19423-s001.jpg]

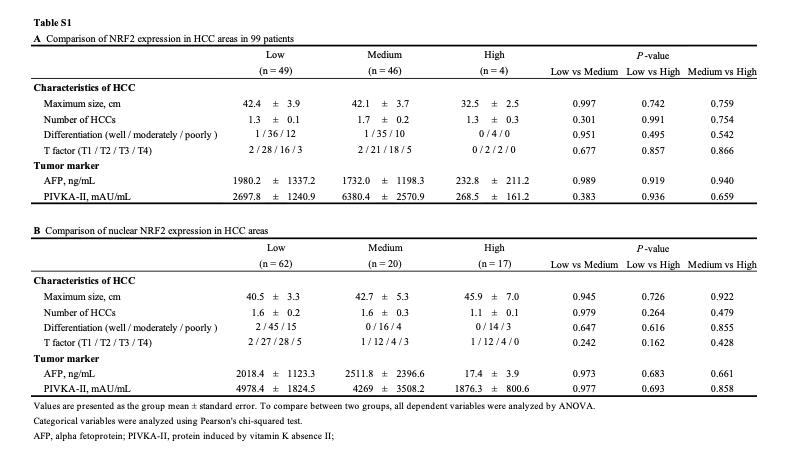

Supplement: Supplementary file 2 — Table S1: [file CAM4-12-19423-s003.jpg]

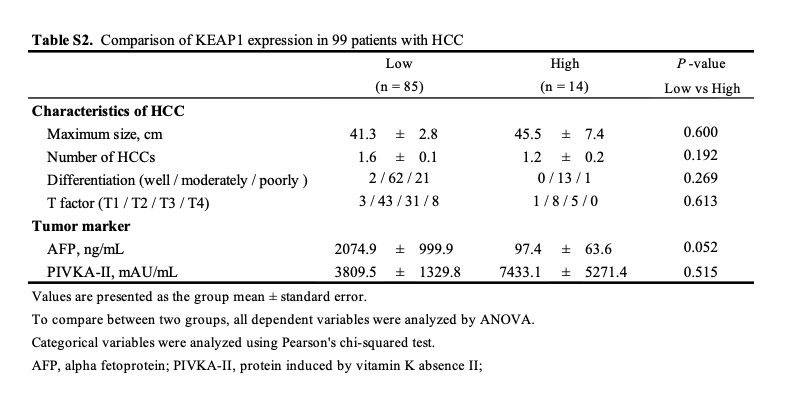

Supplement: Supplementary file 3 — Table S2: [file CAM4-12-19423-s002.jpg]
